# Comparison of short‐term complications after open, laparoscopic and robot‐assisted radical prostatectomy

**DOI:** 10.1111/bju.70076

**Published:** 2025-11-27

**Authors:** Prescillia Nunes, François Richaud, Catherine Quantin, Christine Binquet, Luc Cormier, Anne‐Sophie Mariet

**Affiliations:** ^1^ Université Bourgogne Europe, CHU Dijon Bourgogne Service Biostatistiques et Information Médicale Dijon France; ^2^ Assurance‐Maladie Région Bourgogne‐Franche‐Comté Dijon France; ^3^ Université Bourgogne Europe, CHU Dijon Bourgogne Centre d’Investigation Clinique, Module Epidémiologie Clinique, INSERM, CIC 1432 Dijon France; ^4^ Université Paris‐Saclay UVSQ, INSERM, CESP Villejuif France; ^5^ Department of Urology Dijon‐Bourgogne University Hospital Dijon France

**Keywords:** complications, prostatectomy, robot‐assisted radical prostatectomy, open radical prostatectomy, laparoscopic radical prostatectomy

## Abstract

**Objective:**

The objective of this study is to address the lack of real‐life study comparing the impact of the three surgical approaches for radical prostatectomy (RP), i.e., open (ORP), laparoscopic (LRP) and robot‐assisted (RARP), on the occurrence of postoperative events by measuring the association between surgical approach and risk of death, transfer to an intensive care unit (ICU), or complications during hospitalisation for RP.

**Patients and methods:**

This study used the French National Health Data System (Système National des Données de Santé [SNDS]) to study the 38 481 patients who underwent a RP in French hospitals in 2020–2021. The primary endpoint was the occurrence of any event (death, ICU admission, or complications) during hospitalisation for RP. Secondary endpoints were the occurrence of death, ICU admission, complications, and each of the complication subtypes. Outcomes were analysed by uni‐ and multivariable logistic regression.

**Results:**

A RARP was associated with the lowest risk of an event during hospitalisation, followed by LRP compared with ORP (adjusted odds ratio [aOR] 0.51, 95% confidence interval [CI] 0.48–0.55; and aOR 0.63, 95% CI 0.58–0.68, respectively). RARP was associated with a reduction in the risk of most complications compared with ORP. Minimally‐invasive procedures were associated with an increased risk of hernia. To the best of our knowledge, this is the first French nationwide study of its sort and limitations are related to the observational nature of our study, the use of a medico‐administrative database, and the length of follow‐up. To confirm the main results 30‐day sensitivity analyses were performed.

**Conclusions:**

There were significantly fewer short‐term postoperative events for RARP. Additional studies with a longer follow‐up period are required to investigate the medium‐ and long‐term risks.

**Abbreviations**
CCAM: Classification Commune des Actes Médicaux
CCI: Charlson Comorbidity Index
3D: three‐dimensional
DCIR: Datamart de Consommation Inter‐Regimes
ICD‐10: International Statistical Classification of Diseases and Related Health Problems 10th Revision
ICU: intensive care unit
IQR: interquartile range
LND: lymph node dissection
(a)OR: (adjusted) odds ratio
PMSI: Programme de médicalisation des Systèmes d'Information
RCT: randomised controlled trial
(L)(O)(RA)RP: (laparoscopic) (open) (robot‐assisted) radical prostatectomy
SARS‐Cov‐2: severe acute respiratory syndrome coronavirus 2
SNDS: Système National des Données de Santé

## Introduction

Radical prostatectomy (RP) is one of the reference standard treatments for localised prostate cancer according to national and European guidelines, recommended for patients with a life expectancy of ≥10 years [1, 2].

Historically, RP was performed with a laparotomy or open approach (ORP). In the 1980s, minimally‐invasive procedures appeared with the laparoscopic approach (LRP), and robot‐assisted RP (RARP) has been used since the early 2000s and is now the most common surgical approach for RP in French urological practice [3, 4, 5].

While minimally‐invasive approaches have not demonstrated differences in oncological outcomes, and have longer operating times and higher costs, they offer several advantages such as reduced pain, lower complication rates, faster recovery, better aesthetic results, and improved transfer of technical knowledge [6, 7]. Robot‐assisted surgery has several non‐specific advantages for the surgeon: ergonomic position, eye–hand axis, three‐dimensional (3D) vision, possibility to perform more complex procedures, and high‐quality dissection and suturing thanks to improved manipulation of the instruments [3].

The potential non‐specific and specific complications after RP include death, cardiovascular complications, haemorrhage, injuries, haematoma, anastomotic stenosis, local infection, and urinary retention. It is known that the type and incidence of complications depend on the surgical approach and patient comorbidities [8, 9].

A RARP appears to reduce the overall risk of major complications, which would explain why it is now the most widely used technique. No original studies have compared the three approaches in the same analysis and specifically the risk of complications for each subtype [10, 11, 12].

The primary objective of our study was to estimate the difference in the frequency of adverse events (in‐hospital death, transfer to an intensive care unit [ICU], or complications) during the hospital stay, according to the surgical procedure used for RP (i.e., RARP, LRP, or ORP).

The secondary objectives were to estimate the difference in the frequency of each adverse event and complication separately during the hospitalisation for RP.

## Patients and Methods

### Data Source

The anonymous data were extracted from the French National Health Data System (Système National des Données de Santé [SNDS]), which covers almost the entire French population and contains data relative to health care expenditures reimbursed by national health insurance [13]. Two main databases were linked: the national health insurance reimbursement database (Datamart de Consommation Inter‐Regimes [DCIR]), which contains data for reimbursement of non‐hospital care, and the national hospital database (Programme de médicalisation des Systèmes d'Information [PMSI]), which contains hospital discharge abstracts for all patients admitted to public and private hospitals in France.

Diagnoses and procedures were coded according to the International Statistical Classification of Diseases and Related Health Problems 10th Revision (ICD‐10) and the French Common classification of medical procedures (Classification Commune des Actes Médicaux [CCAM]), respectively.

### Study Design and Population

A nationwide population‐based retrospective cohort study was conducted among men with non‐metastatic prostate cancer treated with RP in 2020 and 2021 in France. Patients with two procedures for RP recorded with inconsistency regarding the surgical approach, the date, or the centre or without robotic assistance specified during laparoscopy (not mandatory before March 2020) were excluded. Patients with a main diagnosis other than prostate cancer or who were suspected of being treated for another condition, considering their CCAM or ICD‐10 codes, were also excluded.

For each patient identified, baseline was defined as the first day of the hospital stay during which the RP procedure was encoded. The hospital stays included interhospital transfers.

### Outcomes

The primary endpoint was composite, defined by the occurrence of at least one of the following adverse events during hospitalisation for RP: in‐hospital death, transfer to an ICU, or complications. Complications were identified by the CCAM codes and ICD‐10 diagnoses encoded during the hospital stay and are detailed in Table 3 and Data S1.

Secondary endpoints were the occurrence of each adverse event: in‐hospital death, transfer to an ICU, overall complications, and each type of complication considered separately.

### Statistical Analysis

The quantitative variables were described as means (SDs) or medians (interquartile range [IQR], Q1–Q3), according to their distribution; qualitative variables were described as numbers and proportions. The characteristics of the three groups were compared using chi‐square for qualitative variables and Kruskal–Wallis test for quantitative variables, in accordance with the conditions of each test.

The association between the surgical approach and the occurrence of postoperative events was analysed using logistic regression models, and crude odds ratios (ORs) and adjusted ORs (aORs) were estimated. Multivariable analyses were performed for the secondary outcomes if the number of events observed was sufficient to estimate the association between the surgical approach and the risk of events taking into account potential confounding factors. Adjustment factors were the variables that can have an effect on the occurrence of events according to clinical knowledge and literature data [14, 15]. They included Charlson Comorbidity Index (CCI), age at entry, hospital type (university hospital, non‐university public hospital, private non‐profit hospital, private hospital), hospital RP volume, severe acute respiratory syndrome coronavirus 2 (SARS‐Cov‐2) infection during hospital stay (ICD‐10 code), and lymph node dissection (LND) during the surgery (CCAM code). Age was included as a categorical variable so that it could be integrated in the same way in all models, as it did not meet the log‐linearity condition for certain secondary endpoints, four classes were defined according to its distribution.

Interactions were explored if suspected according to the literature and results and were integrated in multivariable models if significant. Interactions were tested for each model. To account for alpha risk inflation, we applied the Bonferroni method and considered interactions significant at the 0.0005 threshold, in order to avoid including excessive interactions.

The CCI was calculated using CCAM codes, and diagnoses (ICD‐10 for hospital stays or for Long Duration Disease) and drug reimbursements in the year before hospitalisation for RP [16, 17]. Hospital RP volume was defined as the number of RPs performed in 2019, with categories based on the threshold volumes for urological cancer surgery (30 procedures/year) in France and its multiples.

Length of stay was defined as the time between the date of entry for the hospital stay and the date of discharge, including inter‐hospital transfers.

We performed sensitivity analyses on data collected in the 30 days after RP to confirm the main results. All re‐hospitalisations starting within 30 days after the RP procedure and inter‐hospital transfers were identified and analysed. Stays corresponding to specific and/or iterative treatments (dialysis, chemotherapy, irradiation, brachytherapy, internal irradiation) and stays for cataract surgery were not considered.

Sensitivity analyses were also performed using mixed‐effects logistic regression model with the centre as the random effect to take into account the hierarchy of our data levels and age was considered as a continuous variable if the log‐linearity was respected.

Sensitivity analyses according to whether the annual activity was below or above 15, 30, or 60 RPs performed to confirm that even in centres where activity is very low, the difference between the three surgical approaches exists.

All statistical analyses were performed with the use of Statistical Analysis System (SAS) 9.4 software (SAS Institute, Cary, NC, USA). Statistical significance was set at a *P <* 0.05.

### Ethics

Patient consent was not required, and patient‐identifying information was not used in the research, as this national retrospective study was based on pseudonymised data. The study was conducted according to the guidelines of the Declaration of Helsinki. Prior authorisation from an Ethics Committee was not required following the decree on the SNDS dated 29 June 2021.

## Results

### Study Population Characteristics

**Fig. 1:**
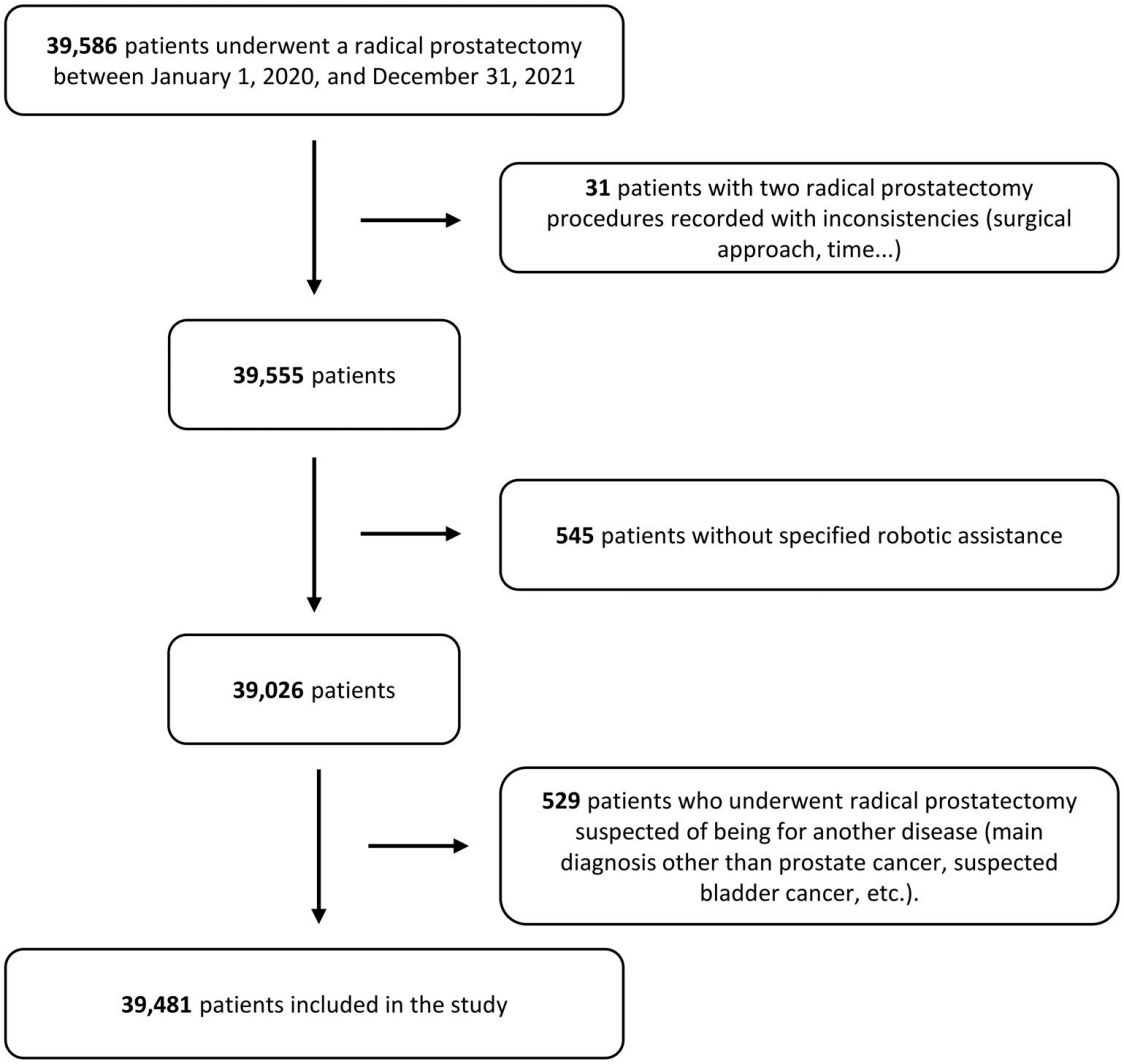
Study population flowchart describing the patients undergoing a RP for non‐metastatic prostate cancer and respecting the other selection criteria (SNDS French national data from 1 January 2020 to 31 December 2021).

Among the 39 586 patients who underwent a RP for non‐metastatic prostate cancer in 2020 and 2021 in France, 38 481 were included (Fig. 1).

The median (IQR) age of the population was 66.0 (61.0–70.0) years, the median (IQR) length of stay was 4.0 (3.0–6.0) days, and 13 387 (34.8%) patients had a CCI of ≥3. Details and patient comorbidities included in the CCI calculation are presented in Table 1.

**Table 1:** Patient characteristics and comorbidities for the entire study population and according to each surgical approach to RP for non‐metastatic prostate cancer – bivariate analyses (SNDS French national data from 1 January 2020 to 31 December 2021, *N* = 38 481).

Variable	Study population	ORP	LRP	RARP	*P*
	38 481 (100%)	7921 (20.6%)	9593 (24.9%)	20 967 (54.5%)	
Age, years					
Mean (SD)	65.2 (6.1)	65.4 (5.9)	65.3 (6.0)	65.1 (6.2)	**0.022**
Median (IQR)	66.0 (61.0–70.0)	66.0 (61.0–70.0)	66.0 (61.0–70.0)	66.0 (61.0–70.0)	
Age (in classes), *n* (%)					
≤61 years	10 011 (26.0)	2015 (25.4)	2447 (25.5)	5549 (26.5)	0.357
62–66 years	10 780 (28.0)	2200 (27.8)	2696 (28.1)	5884 (28.1)	
67–70 years	9745 (25.3)	2052 (25.9)	2452 (25.6)	5241 (25.0)	
>70 years	7945 (20.6)	1654 (20.9)	1998 (20.8)	4293 (20.5)	
CCI score, *n* (%)					
2	25 094 (65.2)	5167 (65.2)	6192 (64.5)	13 735 (65.5)	**<0.001**
3	8190 (21.3)	1712 (21.6)	2165 (22.6)	4313 (20.6)	
≥4	5197 (13.5)	1042 (13.2)	1236 (12.9)	2919 (13.9)	
Comorbidities, *n* (%)					
History of myocardial infarction	2886 (7.5)	533 (6.7)	719 (7.5)	1634 (7.8)	**0.009**
Heart failure	300 (0.8)	61 (0.8)	79 (0.8)	160 (0.8)	0.851
Peripheral vascular disease	1604 (4.2)	332 (4.2)	437 (4.6)	835 (4.0)	0.067
Cerebrovascular disease	919 (2.4)	179 (2.3)	221 (2.3)	519 (2.5)	0.464
Dementia	105 (0.3)	25 (0.3)	29 (0.3)	51 (0.2)	0.469
Chronic pulmonary disease	3732 (9.7)	787 (9.9)	954 (9.9)	1991 (9.5)	0.340
Connective tissue disease	350 (0.9)	72 (0.9)	88 (0.9)	190 (0.9)	0.996
Ulcer disease	146 (0.4)	30 (0.4)	33 (0.3)	83 (0.4)	0.791
Mild liver disease	384 (1.0)	78 (1.0)	104 (1.1)	202 (1.0)	0.610
Moderate/severe liver disease	60 (0.2)	14 (0.2)	13 (0.1)	33 (0.2)	0.787
Diabetes without complication	3832 (10.0)	811 (10.2)	1046 (10.9)	1975 (9.4)	**<0.001**
Diabetes with complication	1063 (2.8)	212 (2.7)	273 (2.8)	578 (2.8)	0.791
Hemiplegia	367 (1.0)	83 (1.0)	85 (0.9)	199 (0.9)	0.545
Moderate/severe kidney disease	877 (2.3)	162 (2.0)	194 (2.0)	521 (2.5)	**0.013**
Other metastatic cancer	1449 (3.8)	284 (3.6)	310 (3.2)	855 (4.1)	**0.001**
HIV (human immunodeficiency virus)	112 (0.3)	8 (0.1)	29 (0.3)	75 (0.4)	**0.001**
Length of stay, days					
Median (IQR)	4.0 (3.0–6.0)	7.0 (5.0–8.0)	4.0 (3.0–7.0)	3.0 (2.0–4.0)	**<0.001**
Year, *n* (%)					
2020	18 049 (46.9)	3926 (49.6)	4599 (47.9)	9524 (45.4)	**<0.001**
2021	20 432 (53.1)	3995 (50.4)	4994 (52.1)	11 443 (54.6)	
Type of facility, *n* (%)					
Private hospital	23 685 (61.5)	5388 (68.0)	6796 (70.8)	11 501 (54.9)	**<0.001**
University hospital	5938 (15.4)	354 (4.5)	834 (8.7)	4750 (22.7)	
Regional hospital	4813 (12.5)	1440 (18.2)	1466 (15.3)	1907 (9.1)	
Non‐profit private hospital	4045 (10.5)	739 (9.3)	497 (5.2)	2809 (13.4)	
Hospital volume (in 2019), *n* (%)					
<30	5762 (15.0)	2783 (35.1)	2095 (21.8)	884 (4.2)	**<0.001**
30–60	10 211 (26.5)	2453 (31.0)	3060 (31.9)	4698 (22.4)	
60–120	14 163 (36.8)	2050 (25.9)	3325 (34.7)	8788 (41.9)	
≥120	8345 (21.7)	635 (8.0)	1113 (11.6)	6597 (31.5)	
LND, *n* (%)					
No	15 405 (40.0)	2029 (25.6)	4079 (42.5)	9297 (44.3)	**<0.001**
Yes	23 076 (60.0)	5892 (74.4)	5514 (57.5)	11 670 (55.7)	
SARS‐Cov‐2 infection during hospital stay, *n* (%)					
No	37 971 (98.7)	7815 (98.7)	9470 (98.7)	20 686 (98.7)	0.913
Yes	510 (1.3)	106 (1.3)	123 (1.3)	281 (1.3)	

Bold values statistically significant at *P* < 0.05.

Overall, 7921 patients (20.6%) underwent an ORP, 9593 (24.9%) underwent a LRP, and 20 967 (54.5%) underwent a RARP (Table 1).

Patient age (in classes) was similar in the three surgical approach groups (*P* = 0.357).

The length of stay was lower for LRP and RARP compared to ORP (*P* < 0.001), with a median (IQR) of 4.0 (3.0–7.0) days, 3.0 (2.0–4.0) days and 7.0 (5.0–8.0) days, respectively (Table 1).

Only 354 (6.0%) RPs were performed with an open procedure in university hospitals vs 1440 (29.9%) in regional hospitals, and 5388 (22.7%) in private hospitals.

In all, 5762 (15.0%) patients underwent RP in a hospital that performed <30 RPs in 2019, and 22 508 (58.5%) patients underwent RP in a centre with a volume of ≥60 RPs.

In all, 73.4% of RARPs were performed in a centre with a volume of ≥60 and 66.1% of ORPs in a centre with a volume <60 (Table 1).

### Outcomes

At least one adverse event occurred during hospitalisation for RP in 6755 (17.6%) patients: 30 (0.1%) patients died, 1059 (2.8%) patients were transferred to ICU, and 6051 (15.7%) patients experienced a postoperative complication (Table 2).

**Table 2:** Occurrence of adverse event during hospital stay for RP for non‐metastatic prostate cancer, in the overall population and according to surgical approach (SNDS French national data from 1 January 2020 to 31 December 2021, *n* = 38 481).

Variable, *n* (%)	Study population	ORP	LRP	RARP
	38 481 (100%)	7921 (20.6%)	9593 (24.9%)	20 967 (54.5%)
**At least one adverse event**	6755 (17.6)	2111 (26.7)	1728 (18.0)	2916 (13.9)
ICU admission	1059 (2.8)	335 (4.2)	241 (2.5)	483 (2.3)
In‐hospital death	30 (0.1)	6 (0.1)	7 (0.1)	17 (0.1)
**Overall complications**	6051 (15.7)	1901 (24.0)	1580 (16.5)	2570 (12.3)
Haemorrhage	2657 (6.9)	835 (10.5)	606 (6.3)	1216 (5.8)
Infection	1718 (4.5)	676 (8.5)	412 (4.3)	630 (3.0)
Hernia	480 (1.2)	66 (0.8)	148 (1.5)	266 (1.3)
Evisceration or eventration	52 (0.1)	13 (0.2)	8 (0.1)	31 (0.1)
Anastomotic leak or fistula	389 (1.0)	111 (1.4)	122 (1.3)	156 (0.7)
Bowel injury	291 (0.8)	69 (0.9)	122 (1.3)	100 (0.5)
Vesical or urethral injury	302 (0.8)	64 (0.8)	111 (1.2)	127 (0.6)
Ureteric injury	211 (0.5)	51 (0.6)	74 (0.8)	86 (0.4)
Vascular injury	93 (0.2)	15 (0.2)	30 (0.3)	48 (0.2)
Other injury	80 (0.2)	19 (0.2)	18 (0.2)	43 (0.2)
Stenosis	103 (0.3)	34 (0.4)	30 (0.3)	39 (0.2)
Nerve lesion	16 (0.0)	4 (0.1)	4 (0.0)	8 (0.0)
Sepsis	92 (0.2)	26 (0.3)	31 (0.3)	35 (0.2)
Embolism or phlebitis	229 (0.6)	92 (1.2)	57 (0.6)	80 (0.4)
Shock	164 (0.4)	56 (0.7)	45 (0.5)	63 (0.3)
Surgical wound dehiscence	280 (0.7)	113 (1.4)	96 (1.0)	71 (0.3)
Lymphocele	306 (0.8)	94 (1.2)	77 (0.8)	135 (0.6)
Foreign bodies	8 (0.0)	4 (0.1)	3 (0.0)	1 (0.0)
Urinary retention	436 (1.1)	163 (2.1)	147 (1.5)	126 (0.6)

#### Primary Outcome

The frequency of adverse events during the hospital stay was lowest for RARP with 2916 (13.9%) patients, followed by LRP with 1728 (18.0%), and then ORP with 2111 (26.7%) patients (*P* < 0.001; Table 2 and S1). Thus, patients who underwent RARP and LRP had a lower risk of a postoperative event than those who underwent ORP, and RARP appeared to be the surgical approach with the lowest risk of adverse events (aOR 0.63, 95% CI 0.58–0.68 for LRP and aOR 0.51, 95% CI 0.48–0.55 for RARP; Table 3). The 30‐day findings were similar to the main results, with a reduction of risk of adverse events by 39% for RARP (OR 0.61, 95% CI 0.57–0.65) and 30% for LRP compared to ORP (aOR 0.70, 95% CI 0.66–0.75).

**Table 3:** Association between the surgical approach and the occurrence of adverse event during hospital stay for RP for non‐metastatic prostate cancer (SNDS French national data from 1 January 2020 to 31 December 2021, *N* = 38 481), univariable and multivariable analyses.

Variable	Yes, *n* (%)	No, *n* (%)	Crude OR (95% CI)	*P*	Adjusted OR (95% CI)*	*P*
**At least one adverse event** ^§^						
ORP	2111 (26.7)	5810 (73.3)	REF.	**<0.001**	REF.	**<0.001**
LRP	1728 (18.0)	7865 (82.0)	0.61 (0.56–0.65)		0.63 (0.58–0.68)	
RARP	2916 (13.9)	18 051 (86.1)	0.44 (0.42–0.47)		0.51 (0.48–0.55)	
**ICU admission** ^§^ ^,^ ^¶^						
ORP	335 (4.2)	7586 (95.8)	REF.	**<0.001**	REF.	**<0.001**
LRP	241 (2.5)	9352 (97.5)	0.58 (0.49–0.69)		0.62 (0.52–0.73)	
RARP	483 (2.3)	20 484 (97.7)	0.53 (0.46–0.62)		0.81 (0.70–0.95)	
**Overall complications** ^§^						
ORP	1901 (24.0)	6020 (76.0)	REF.	**<0.001**	REF.	**<0.001**
LRP	1580 (46.5)	8013 (83.5)	0.62 (0.58–0.67)		0.65 (0.60–0.70)	
RARP	2570 (12.3)	18 397 (87.7)	0.44 (0.41–0.47)		0.48 (0.45–0.52)	

* Multivariable logistic regressions adjusted on age group, CCI category, hospital type, hospital volume, SARS‐Cov‐2 infection during hospital stay, and LND.

^§^ Interaction between hospital type and hospital volume.

^¶^ Interaction between hospital volume and LND.

Bold values statistically significant at *P* < 0.05.

#### Secondary Outcomes

The risk of in‐hospital death was comparable between the three groups (*P* = 0.970; Table 2 and S1).

The risk of ICU transfer was associated with surgical approach (*P* < 0.001), with reductions of 19% (aOR 0.81, 95% CI 0.70–0.95) for RARP and 39% (aOR 0.61, 95% CI 0.52–0.73) for LRP compared to ORP. There was no significant difference between RARP and LRP (Table 3).

**Fig. 2:**
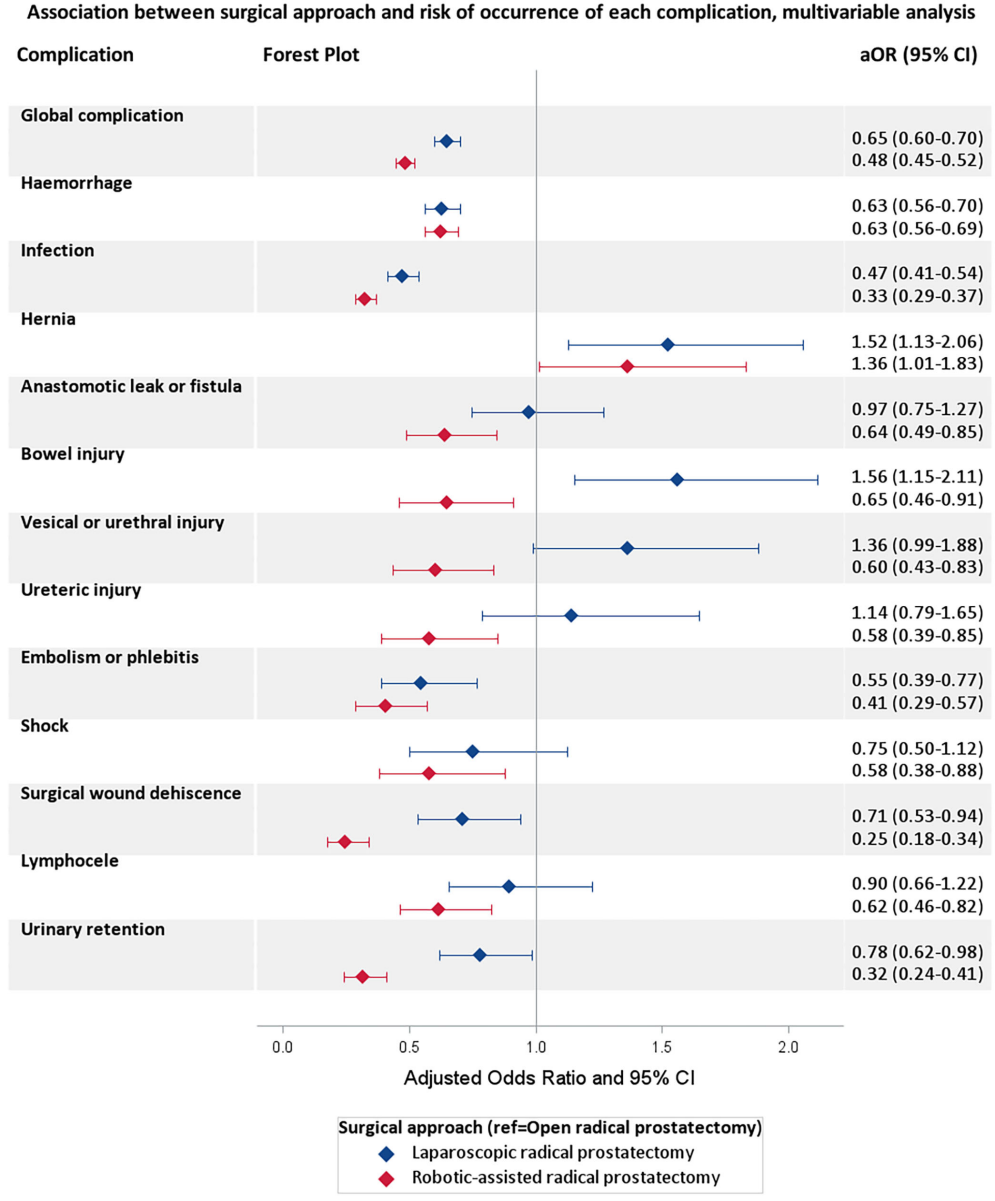
Association between the surgical approach and the occurrence of complications during the hospital stay for RP (LRP and RARP vs LRP) in patients with non‐metastatic prostate cancer (SNDS French national data from 1 January 2020 to 31 December 2021, *N* = 38 481) multivariable logistic regressions.

Both RARP and LRP were associated with a lower risk of overall complications (*P* < 0.001), but the reduction was bigger for RARP than for LRP (aOR 0.48, 95% CI 0.45–0.52 and aOR 0.65, 95% CI 0.60–0.70, respectively; Fig. 2, Tables 3 and S1).

Haemorrhage (10.5% for ORP, 6.3% for LRP, 5.8% for RARP) and infection (8.5% for ORP, 4.3% for LRP, and 3.0% for RARP) were the two main complications for the three surgical approaches (Table 2). These results were confirmed by sensitivity analyses at 30 days (Table S2).

Compared to ORP, RARP was associated with a lower risk of most complication subtypes: haemorrhage, infection, anastomotic leak or fistula, bowel injury, vesical or urethral injury, ureteric injury, embolism or phlebitis, shock, surgical wound dehiscence, lymphocele, and urinary retention (Table S1). At 30 days we observed the same results except for the risk of anastomotic leak or fistula, bowel injury, and shock (Table S3).

The LRP was associated with a lower risk of haemorrhage, infection, embolism or phlebitis, surgical wound dehiscence, and urinary retention than ORP, but a higher risk of bowel injury (Fig. 2 and Table S1). At 30 days we observed the same results except for the risk of urinary retention (Table S3).

Both LRP and RARP were associated with an increased risk of hernia (aOR 1.52, 95% CI 1.13–2.06 and aOR 1.36, 95% CI = 1.01–1.83, respectively; Fig. 2 and Table S1). Similar results were observed after the sensitivity analyses at 30‐days (Tables S2 and S3), but the results were not confirmed in the mixed‐effects logistic regression model, when the effect of the centre was taken into account, in both analyses during the hospital stay or at 30‐days (Tables S4 and S5).

Taking into account the centre effect in the models and integrating age as a continuous variable confirm our main results. However, taking into account the centre effect modifies the estimates of the effect of the surgical approach on the risks of some secondary endpoints during the hospital stay (other than hernia): the risk of ICU admission was not still significantly associated with the surgical approach, minimally‐invasive surgeries were associated with a higher risk of vesical or urethral injury (*P* < 0.001), the risk of lymphocele was no more decreased by RARP compared to ORP, and the risk of urinary retention no more decreased by urinary retention (Table S4). Results of mixed‐effects logistic regression models at 30 days were also available in Table S5.

A LND was most frequently performed when ORP was performed. The descriptions of each complication by subgroup are available in Tables S6 and S7.

Subgroup analyses according to whether annual activity was below or above 15, 30, or 60 RPs are presented in Tables S8–, S10. Subgroups operated on in low‐volume centres generally had more events, and for each subgroup, we found differences between the three surgical approaches, with ORP having the most complications, followed by LRP and RARP.

## Discussion

To the best of our knowledge, this is the first French nationwide study of its sort. We included nearly 40 000 patients who underwent RP over a 2‐year period, comparing the risk of different complications according to the surgical approach (ORP, LRP, and RARP), identifying complications using routinely coded diagnoses and procedures, and taking comorbidities into account.

### Results and Comparison with Other Studies

This study found that 20% of RPs were still performed by laparotomy, while almost a quarter were done by laparoscopy, and more than half were performed with robotic assistance. Our results confirm that RARP has replaced ORP as the most used technique in France, similar to other developed countries in Europe and the United States [18]. However, they also reveal the French particularity of maintaining a high proportion of LRPs.

The length of hospital stay depends on the social context. Hospital stays are longer in France than in many other countries [19]. However, even if French habits differ from those of other countries, there are differences between the three surgical techniques, with a shorter length of stay for RARP. Our results are consistent with the results of another French study and several systematic analyses [10, 12, 20, 21].

This observation makes the duration of exposure to in‐hospital complications different between the groups. The aim of the 30‐day analyses was to define an equivalent observation time between the compared groups to ensure that our results were not biased by this element. The results of these sensitivity analyses confirmed our findings. Our results suggest that the surgical technique seems to have more effect on the risk of complications during the hospital stay than in the 30‐day period following the surgery.

Our main results are inconsistent with those of a meta‐analysis published in 2019, which concluded that no surgical approach was superior in terms of postoperative complications or functional and oncological outcomes, but the heterogeneity of the studies and their small number weakened the level of evidence [21]. However, they are consistent with the results of randomised controlled trials (RCTs) and a meta‐analysis of RCTs, which showed the superiority of RARP [7, 22, 23]. These published RCT studies are monocentric, with small numbers of patients included compared to our study, and with the selection biases that this experimental design implies due to the inclusion and exclusion criteria required. Moreover, these studies only compare RARP vs ORP.

Complication rates are heterogeneous in the literature, depending on the data used. Our rates are either consistent with [11] or higher than reports in previous studies [10, 20]. Ploussard et al. [10] analysed postoperative outcomes using PMSI data, but the complication rate was estimated using a severity index taking into account both comorbidities and complications, rather than using diagnoses and procedures performed during hospitalisation. However, RARP was associated with a lower risk of postoperative complications both in our study and in theirs [10, 12].

The risk of haemorrhage appeared to be lower for RARP and LRP than ORP, and it did not differ between RARP and LRP. Our definition of haemorrhage included transfusion procedures and diagnoses encoded during the hospitalisation, while other authors evaluated transfusion rates and blood loss. However, our results are similar to other studies, indicating that there is a lower transfusion risk for RARP and LRP than for ORP. Basiri et al. [12] showed no difference while Tewari et al. [20] found a higher transfusion risk for LRP than RARP. Also, they observed less blood loss for RARP and LRP compared to ORP and more blood loss for LRP than RARP [12, 20, 21, 24].

The risk of infection and surgical wound dehiscence appeared to be lowest for RARP, followed by LRP and ORP. To our knowledge, this is the first time that the risk of surgical wound dehiscence was compared between the three approaches. The risk of infection in our study included digestive, genitourinary, and wound infection, complicating comparisons with other studies that only evaluated the risk of wound infection, which appeared to be lower for both minimally‐invasive procedures than for ORP, but comparable between RARP and LRP [20].

The risk of bowel injuries was higher for LRP than RARP and ORP, which is consistent with the literature [24]. This could be due to the reduced articulation of the instruments with LRP compared to ORP. With the introduction of RARP, the surgeon was again able to articulate the instruments while taking advantage of the pneumoperitoneum induced for mini‐invasive procedures and improved visualization of the organs with 3D vision.

The RARP appeared to be associated with a reduction of anastomotic leak or fistula and urinary retention, compared to ORP. Codes used do not allow us to distinguish between rectourethral fistulae and anastomotic leaks, but it is known that fistulae rarely occur during the perioperative period (during hospital stay), so most of the cases identified are likely to be anastomotic leaks. These observations can be explained by the fact that robotic assistance seems to improve the quality of the vesico‐urethral anastomosis. However, the difference of the risk of anastomotic leak or fistula is not confirmed with longer follow‐up (30 days). Tewari et al. [20] found a higher rate of fistula for LRP than ORP and RARP, and no difference between RARP and ORP, during the perioperative period, defined as the first 30 days following surgery.

Previous studies have also found that more LNDs are performed in open surgery [25]. Our results were adjusted on this information, and we found no interaction between LND and surgical approach for the overall risk of adverse events, meaning that the association between surgical approach and event risk did not differ depending on whether LND was performed or not.

There was a significant association between the risk of hernia and the surgical approach: the risk appeared to be increased by RARP and LRP. This finding is contrary to other studies that found a higher incidence of hernia after ORP than RARP and LRP, and no difference between RARP and LRP [26]. Our observation reflects the use of prolonged pneumoperitoneum at relatively high pressure with minimally‐invasive approaches. Moreover, unlike ORP, minimally‐invasive RP is an intraperitoneal intervention requiring the release of the hernial orifices from the peritoneum, which could explain the increased risk of hernia in our study. However, these results were not confirmed when the effect of the centre was taken into account.

As reflected in our results, it is already known that the risk of lymphocele is strongly related to LND [27]. The surgical approach was significantly associated with a reduction of the risk when RARP is performed, whether LND was performed or not.

Our results reveal that RARP was more likely than ORP to be performed in a university hospital (22.7% vs 4.5%) than in a regional facility (9.1% vs 18.3%). This means that RARP is performed in more major centres with potentially more experienced surgeons and more advanced perioperative pathways. Major tertiary and university hospitals have registrars and urological training fellows as well as experienced nursing staff that can generally manage postoperative complications on site, whereas smaller regional hospitals or private hospitals may require a similar patient to be transferred to an ICU facility. However, after adjusting for interactions with the type of centre there was still a difference between surgical techniques.

In addition, we found that a higher proportion of HIV‐positive patients underwent minimally‐invasive surgery than ORP. Importantly, these approaches generally involve less risk of exposure to biological fluids, making them a safer alternative for the surgeon and operating room staff.

### Strength and Limitations

Our study, using real‐life data, offers advantages such as the exhaustive inclusion of all centres in France that practice RP, all patients treated in these centres, as well as the possibility of comparing the three techniques (RARP, LRP, and ORP) bringing a complementary approach to already published studies. In addition, our study analysed complications using routinely coded diagnoses and procedures and taking into account comorbidities identified during the year prior to surgery, by cross‐referencing medico‐administrative data, in order to offer a higher level of detail and account for most confounding factors.

Biases related to under‐detection or misclassification need to be taken into account, particularly for comorbidities and complications, even if they are very likely to be non‐differential between the three groups. Because funding for all care expenditures (non‐hospital and hospital) is based on the SNDS, collected data are tightly controlled, guaranteeing high standards of coherence, accuracy, and exhaustiveness.

Although we have taken confounding factors into account, we cannot guarantee the absence of residual bias related to unmeasured confounding factors, which may be due to the absence of clinical data given the nature of our data and the possibility of unknown confounding factors.

There is a risk of bias linked to conversions that cannot be identified because of coding rules. However, the risk of conversion is low, as highlighted in the literature and as experience has shown [28, 29]. Although this low risk creates a potential bias against ORP, it does not seem to us to explain all the difference observed between the minimally‐invasive procedures and ORP.

There is a potential for classification biases in the comorbidity index because we could not check patient medical records. However, calculations were made using validated algorithms [16, 17].

Our data contained no details regarding the characteristics of the tumour, but RP is indicated for patients with localised prostate cancer, and we limited our investigation on patients undergoing surgical management for prostate cancer with no other oncological surgical treatments.

Our study used a database to evaluate complications that occurred during hospitalisation for RP. We were unable to access patient medical records, which limited our ability to assess functional outcomes. Therefore, we could not confirm results from other studies suggesting that RARP improves urinary continence and erectile function [11, 24, 30, 31].

Our data source contained no details regarding the RARP procedure (transperitoneal or extraperitoneal route/insertion of camera port/closing technique/closure by nurse or intern and/or attending/consultant). These details would be of interest to better understand how and why minimally‐invasive surgeries increase the risk of hernia. It would be interesting to analyse this information to better understand and learn from RARP, which is becoming a dominant approach.

It is recognised that the surgeon's experience has an impact on postoperative outcomes after RP [32]. Unfortunately, information regarding surgeon volume was not available in our data source. However, in order to approximate surgeon experience, and because team experience is also important, we adjusted our models on volume of RPs by centre. Moreover, subgroup analyses based on whether annual activity was below or above 15, 30, or 60 RPs showed that even in centres with very low activity, while the risk of complications increased as annual activity decreased, a difference between surgical approaches persisted. Indeed, in other surgeries, it has been shown that low‐volume surgeons do not achieve different results from their high‐volume counterparts when practicing in a high‐volume facility. This means that, although volume per surgeon is important, there is a ‘field effect’ that makes hospital volume important in the discussion [33]. The year 2019 was chosen as the reference year to limit the impact of the health crisis on this variable. However, it should be noted that even though our study period was during the COVID‐19 crisis, French surgical oncology activity was not seriously disrupted [34].

With the large power of our study, significant differences may be found for small variations. However, the details by complication subtype are presented for exploratory purposes and should be interpreted with caution. Only ICU admission, haemorrhage, infection, and urinary retention differed by >1% (one per 100 patients) between procedures. Nevertheless, the differences observed were substantial for our primary endpoint, and for the overall complication risk.

Thus, although there were strong associations between postoperative events during hospitalisation and the surgical approach in our study, taking into account several adjustment variables, our study could not establish a causal relationship, and the causal pathways require further evaluation.

### Perspectives

This study compared the risk of short‐term events, but further research is needed to evaluate medium‐ and long‐term outcomes using in‐hospital and out‐of‐hospital data from the SNDS.

We are also awaiting the results of an economic evaluation of RARP compared to LRP and ORP based on the SNDS data [35].

## Conclusions

In conclusion, this nationwide study confirmed that surgical approach (RARP, LRP, or ORP) was independently associated with the risk of short‐term postoperative events after RP, after adjusting for age, CCI, hospital type, hospital volume, SARS‐Cov‐2 infection, and LND. The risk was lower for RARP and LRP compared to ORP in our study. The risk of an in‐hospital event after RARP was close to two‐fold lower than after ORP, and the reduction of this risk was higher after RARP than LRP. Considering that an open surgical approach is still used in 20% of RPs, this study highlights the benefits of the RARP on in‐hospital adverse events and on complications during the hospital stay.

## Disclosure of Interests

The authors declare no conflicts of interest associated with this publication and no significant financial support for this work that could have influenced its outcomes.

## Supporting information


**Table S1.** Association between the surgical approach and the occurrence of each subtype of adverse event during hospital stay for RP for non‐metastatic prostate cancer (SNDS French national data from 1 January 2020 to 31 December 2021, *N* = 38 481), uni‐ and multivariable analyses.

**Table S2.** Occurrence of adverse event during initial hospital stay for RP for non‐metastatic prostate cancer and re‐hospitalisations starting within the 30 days after RP, in the overall population and according to surgical approach (SNDS French national data from 1 January 2020 to 31 December 2021, *N* = 38 481).

**Table S3.** Association between the surgical approach and the occurrence of each subtype of adverse event during the initial hospital stay for RP for non‐metastatic prostate cancer and re‐hospitalisations starting within the 30 days after RP (SNDS French national data from 1 January 2020 to 31 December 2021, *N* = 38 481), uni‐ and multivariable analyses.

**Table S4.** Association between the surgical approach and the occurrence of each subtype of adverse event during the initial hospital stay for RP for non‐metastatic prostate cancer (SNDS French national data from 1 January 2020 to 31 December 2021, *N* = 38 481), mixed‐effects logistic regression models.

**Table S5.** Association between the surgical approach and the occurrence of each subtype of adverse event during the initial hospital stay for RP for non‐metastatic prostate cancer and re‐hospitalisations starting within the 30 days after RP (SNDS French national data from 1 January 2020 to 31 December 2021, *N* = 38 481), mixed‐effects logistic regression models.

**Table S6.** Subgroup description of the occurrence of adverse event during hospital stay for RP for non‐metastatic prostate cancer, in the overall population and according to surgical approach (SNDS French national data from 1 January 2020 to 31 December 2021, *N* = 38 481).

**Table S7.** Subgroup description of the occurrence of adverse event during initial hospital stay for RP for non‐metastatic prostate cancer and re‐hospitalisations starting within the 30 days after RP, in the overall population and according to surgical approach (SNDS French national data from 1 January 2020 to 31 December 2021, *N* = 38 481).

**Table S8.** Subgroup description of the occurrence of adverse event during initial hospital stay for RP for non‐metastatic prostate cancer, according to the annual activity of RP in the establishment (SNDS French national data from 1 January 2020 to 31 December 2021, *N* = 38 481).

**Table S9.** Subgroup description of the occurrence of adverse event during initial hospital stay for RP for non‐metastatic prostate cancer, according to the annual activity of RP in the establishment (SNDS French national data from 1 January 2020 to 31 December 2021, *N* = 38 481).

**Table S10.** Subgroup description of the occurrence of adverse event during initial hospital stay for RP for non‐metastatic prostate cancer, according to the annual activity of RP in the establishment (SNDS French national data from 1 January 2020 to 31 December 2021, *N* = 38 481).

**Data S1.** This file contains details on the CCAM codes and ICD‐10 diagnoses encoded during the hospital stay and used to identify complications.
