# Supplementary material for: Comparison of short‐term complications after open, laparoscopic and robot‐assisted radical prostatectomy
Source: BJU Int. 2025 Nov 27;137(2):348–59. doi: 10.1111/bju.70076 (PMC12789849; doi:10.1111/bju.70076)
Supplement: Supplementary file 1 — Table S1. Association between the surgical approach and the occurrence of each subtype of adverse event during hospital stay for RP for non‐metastatic prostate cancer (SNDS French national data from 1 January 2020 to 31 December 2021, N = 38 481), uni‐ and multivariable analyses. [file BJU-137-348-s003.pdf]

Supplementary table 1. Association between the surgical approach and the occurrence of each subtype of adverse event during hospital stay for radical prostatectomy for non-metastatic prostate cancer (SNDS French national data from January 1, 2020, to December 31, 2021, n=38,481), univariable and multivariable analyses

	Odds ratio (95% CI)	p	Adjusted Odds ratio (95% CI) *	p
**At least one adverse event (n=6,755)** [3]		**<.0001**		**<.0001**
ORP	REF.		REF.	
LRP	0.61 (0.56-0.65)		0.63 (0.58-0.68)	
RARP	0.44 (0.42-0.47)		0.51 (0.48-0.55)	
**Intensive care unit admission (n=1,059)** [3 4]		**<.0001**		**<.0001**
ORP	REF.		REF.	
LRP	0.58 (0.49-0.69)		0.61 (0.52-0.73)	
RARP	0.53 (0.46-0.62)		0.81 (0.70-0.95)	
**In-hospital death (n=30)**		0.9696		**-**
ORP	REF.		-	
LRP	0.96 (0.32-2.87)		-	
RARP	1.07 (0.42-2.72)		-	
**Overall complications (n=6,051)** [3]		**<.0001**		**<.0001**
ORP	REF.		REF.	
LRP	0.62 (0.58-0.67)		0.65 (0.60-0.70)	
RARP	0.44 (0.41-0.47)		0.48 (0.45-0.52)	
**Haemorrhage (n=2,657)** [3]		**<.0001**		**<.0001**
ORP	REF.		REF.	
LRP	0.57 (0.51-0.64)		0.63 (0.56-0.70)	
RARP	0.52 (0.48-0.57)		0.63 (0.56-0.69)	
**Infection (n=1,718)** [3]		**<.0001**		**<.0001**
ORP	REF.		REF.	
LRP	0.48 (0.42-0.55)		0.47 (0.41-0.54)	
RARP	0.33 (0.30-0.37)		0.33 (0.29-0.37)	
**Hernia (n=480)** [1]		**0.0002**		**0.0222**
ORP	REF.		REF.	
LRP	1.87 (1.39-2.50)		1.52 (1.13-2.06)	
RARP	1.53 (1.17-2.00)		1.36 (1.01-1.83)	
**Evisceration or eventration (n=52)**		0.2769		**-**
ORP	REF.		-	
LRP	0.51 (0.21-1.23)		-	
RARP	0.90 (0.47-1.72)		-	
**Anastomotic leak or fistula (n=389)**		**<.0001**		**0.0013**
ORP	REF.		REF.	
LRP	0.91 (0.70-1.17)		0.97 (0.75-1.27)	

RARP	0.53 (0.41-0.67)		0.64 (0.49-0.85)	
**Bowel injury (n=291)**		**<.0001**		**<.0001**
ORP	REF.		REF.	
LRP	1.47 (1.09-1.97)		1.56 (1.15-2.11)	
RARP	0.55 (0.40-0.74)		0.65 (0.46-0.91)	
**Vesical or urethral injury (n=302)** [1 3 4]		**<.0001**		**<.0001**
ORP	REF.		REF.	
LRP	1.44 (1.06-1.96)		1.36 (0.99-1.88)	
RARP	0.75 (0.55-1.01)		0.60 (0.43-0.83)	
**Ureteric injury (n=211)**		**0.0002**		**0.0002**
ORP	REF.		REF.	
LRP	1.20 (0.84-1.72)		1.14 (0.79-1.65)	
RARP	0.64 (0.45-0.90)		0.58 (0.39-0.85)	
**Vascular injury (n=93)**		0.2220		**-**
ORP	REF.		-	
LRP	1.65 (0.89-3.08)		-	
RARP	1.21 (0.68-2.16)		-	
**Other injury (n=80)**		0.7458		**-**
ORP	REF.		-	
LRP	0.78 (0.41-1.49)		-	
RARP	0.85 (0.50-1.47)		-	
**Stenosis (n=103)**		**0.0014**		**-**
ORP	REF.		-	
LRP	0.73 (0.44-1.19)		-	
RARP	0.43 (0.27-0.69)		-	
**Sepsis (n=92)**		**0.0078**		**-**
ORP	REF.		-	
LRP	0.98 (0.58-1.66)		-	
RARP	0.51 (0.31-0.84)		-	
**Embolism of phlebitis (n=229)**		**<.0001**		**<.0001**
ORP	REF.		REF.	
LRP	0.51 (0.37-0.71)		0.55 (0.39-0.77)	
RARP	0.33 (0.24-0.44)		0.41 (0.29-0.57)	
**Shock (n=164)**		**<.0001**		**0.0359**
ORP	REF.		REF.	
LRP	0.66 (0.45-0.98)		0.75 (0.50-1.12)	
RARP	0.42 (0.30-0.61)		0.58 (0.38-0.88)	
**Surgical wound dehiscence (n=280)**		**<.0001**		**<.0001**
ORP	REF.		REF.	
LRP	0.70 (0.53-0.92)		0.71 (0.53-0.94)	
RARP	0.23 (0.17-0.32)		0.25 (0.18-0.34)	
**Lymphocele (n=306)** [3]		**<.0001**		**0.0027**
ORP	REF.		REF.	
LRP	0.67 (0.50-0.91)		0.90 (0.66-1.22)	

RARP	0.54 (0.41-0.70)		0.62 (0.46-0.82)	
**Urinary retention (n=436)**		**<.0001**		**<.0001**
ORP	REF.		REF.	
LRP	0.74 (0.59-0.93)		0.78 (0.62-0.98)	
RARP	0.29 (0.23-0.36)		0.32 (0.24-0.41)	

* Multivariable logistic regressions adjusted on age group, Charlson comorbidity index category, hospital type, hospital volume, Sars-Cov2 infection during hospital stay and lymph node dissection

[1] interaction between lymph node dissection and hospital type; [2] interaction between lymph node dissection and Charlson comorbidity index category; [3] interaction between hospital type and hospital volume; [4] interaction between hospital volume and lymph node dissection

ORP = Open radical prostatectomy / LRP = Laparoscopic radical prostatectomy / RARP = Robot-assisted radical prostatectomy
