# Supplementary material for: Comparison of short‐term complications after open, laparoscopic and robot‐assisted radical prostatectomy
Source: BJU Int. 2025 Nov 27;137(2):348–59. doi: 10.1111/bju.70076 (PMC12789849; doi:10.1111/bju.70076)
Supplement: Supplementary file 2 — Table S2. Occurrence of adverse event during initial hospital stay for RP for non‐metastatic prostate cancer and re‐hospitalisations starting within the 30 days after RP, in the overall population and according to surgical approach (SNDS French national data from 1 January 2020 to 31 December 2021, N = 38 481). [file BJU-137-348-s011.pdf]

Supplementary table 2. Occurrence of adverse event during initial hospital stay for radical prostatectomy for non-metastatic prostate cancer and re-hospitalizations starting within the 30 days after radical prostatectomy, in the overall population and according to surgical approach (SNDS French national data from January 1, 2020, to December 31, 2021, n=38,841)

	**Study population**	**ORP 7,921 (20.6%)**	**LRP 9,593 (24.9%)**	**RARP 20,967 (54.5%)**
**At least one adverse event**	8,961 (23.3%)	2,498 (31.5%)	2,266 (23.6%)	4,197 (20.0%)
**ICU admission**	1,162 (3.0%)	351 (4.4%)	261 (2.7%)	550 (2.6%)
**In-hospital death**	43 (0.1%)	7 (0.1%)	11 (0.1%)	25 (0.1%)
**Overall complications**	8,296 (21.6%)	2,295 (29.0%)	2,129 (22.2%)	3,872 (18.5%)
Haemorrhage	3,341 (8.7%)	954 (12.0%)	752 (7.8%)	1,635 (7.8%)
Infection	3,023 (7.9%)	920 (11.6%)	758 (7.9%)	1,345 (6.4%)
Hernia	508 (1.3%)	69 (0.9%)	153 (1.6%)	286 (1.4%)
Evisceration or eventration	116 (0.3%)	18 (0.2%)	13 (0.1%)	85 (0.4%)
Anastomotic leak or fistula	683 (1.8%)	152 (1.9%)	204 (2.1%)	327 (1.6%)
Bowel injury	331 (0.9%)	73 (0.9%)	131 (1.4%)	127 (0.6%)
Vesical or urethral injury	382 (1.0%)	74 (0.9%)	139 (1.4%)	169 (0.8%)
Ureteric injury	281 (0.7%)	63 (0.8%)	89 (0.9%)	129 (0.6%)
Vascular injury	141 (0.4%)	29 (0.4%)	38 (0.4%)	74 (0.4%)
Other injury	127 (0.3%)	26 (0.3%)	27 (0.3%)	74 (0.4%)
Stenosis	154 (0.4%)	52 (0.7%)	45 (0.5%)	57 (0.3%)
Nerve lesion	18 (0.0%)	4 (0.1%)	4 (0.0%)	10 (0.0%)
Sepsis	179 (0.5%)	40 (0.5%)	53 (0.6%)	86 (0.4%)
Embolism or phlebitis	350 (0.9%)	116 (1.5%)	87 (0.9%)	147 (0.7%)
Shock	228 (0.6%)	67 (0.8%)	59 (0.6%)	102 (0.5%)
Surgical wound dehiscence	447 (1.2%)	168 (2.1%)	124 (1.3%)	155 (0.7%)
Lymphocele	612 (1.6%)	175 (2.2%)	164 (1.7%)	273 (1.3%)
Foreign bodies	9 (0.0%)	4 (0.1%)	3 (0.0%)	2 (0.0%)
Urinary retention	1,175 (3.1%)	304 (3.8%)	352 (3.7%)	519 (2.5%)

ORP = Open radical prostatectomy / LRP = Laparoscopic radical prostatectomy / RARP = Robot-assisted prostatectomy / ICU = Intensive Care Unit
