# Supplementary material for: Comparison of short‐term complications after open, laparoscopic and robot‐assisted radical prostatectomy
Source: BJU Int. 2025 Nov 27;137(2):348–59. doi: 10.1111/bju.70076 (PMC12789849; doi:10.1111/bju.70076)
Supplement: Supplementary file 3 — Table S3. Association between the surgical approach and the occurrence of each subtype of adverse event during the initial hospital stay for RP for non‐metastatic prostate cancer and re‐hospitalisations starting within the 30 days after RP (SNDS French national data from 1 January 2020 to 31 December 2021, N = 38 481), uni‐ and multivariable analyses. [file BJU-137-348-s004.pdf]

Supplementary table 3. Association between the surgical approach and the occurrence of each subtype of adverse event during the initial hospital stay for radical prostatectomy for non-metastatic prostate cancer and re-hospitalizations starting within the 30 days after radical prostatectomy (SNDS French national data from January 1, 2020, to December 31, 2021, n=38,481), univariable and multivariable analyses

	Odds ratio (95% CI)	p	Adjusted Odds ratio (95% CI) *	p
**At least one adverse event (n=8,961)** [3 4]		**<.0001**		**<.0001**
ORP	REF.		REF.	
LRP	0.67 (0.63-0.72)		0.70 (0.66-0.75)	
RARP	0.54 (0.51-0.58)		0.61 (0.57-0.65)	
**Intensive care unit admission (n=1,162)** [3]		**<.0001**		**<.0001**
ORP	REF.		REF.	
LRP	0.60 (0.51-0.71)		0.64 (0.54-0.75)	
RARP	0.58 (0.51-0.67)		0.85 (0.73-0.99)	
**In-hospital death (n=43)**		0.7804		-
ORP	REF.		-	
LRP	1.30 (0.50-3.35)		-	
RARP	1.35 (0.58-3.12)		-	
**Overall complications (n=8,296)** [3]		**<.0001**		**<.0001**
ORP	REF.		REF.	
LRP	0.70 (0.65-0.75)		0.73 (0.68-0.79)	
RARP	0.56 (0.52-0.59)		0.60 (0.56-0.64)	
**Haemorrhage (n=3,341)** [3]		**<.0001**		**<.0001**
ORP	REF.		REF.	
LRP	0.62 (0.56-0.69)		0.67 (0.60-0.74)	
RARP	0.62 (0.57-0.67)		0.70 (0.63-0.77)	
**Infection (n=3,023)** [3]		**<.0001**		**<.0001**
ORP	REF.		REF.	
LRP	0.65 (0.59-0.72)		0.66 (0.60-0.73)	
RARP	0.52 (0.48-0.57)		0.53 (0.48-0.58)	
**Hernia (n=508)** [1]		**0.0001**		**0.0157**
ORP	REF.		REF.	
LRP	1.84 (1.39-2.45)		1.53 (1.14-2.06)	
RARP	1.57 (1.21-2.05)		1.42 (1.06-1.89)	
**Evisceration or eventration (n=116)**		**0.0003**		**-**
ORP	REF.		-	
LRP	0.60 (0.29-1.22)		-	
RARP	1.79 (1.07-2.97)		-	
**Anastomotic leak or fistula (n=683)** [3]		**0.0013**		**0.0211**

ORP	REF.		REF.	
LRP	1.11 (0.90-1.37)		1.21 (0.97-1.50)	
RARP	0.81 (0.67-0.98)		0.92 (0.74-1.15)	
**Bowel injury (n=331)**		**<.0001**		**<.0001**
ORP	REF.		REF.	
LRP	1.49 (1.12-1.98)		1.62 (1.20-2.17)	
RARP	0.66 (0.49-0.87)		0.80 (0.58-1.09)	
**Vesical or urethral injury (n=382)** [1 3 4]		**<.0001**		**<.0001**
ORP	REF.		REF.	
LRP	1.56 (1.17-2.07)		1.46 (1.09-1.96)	
RARP	0.86 (0.65-1.13)		0.68 (0.50-0.92)	
**Ureteric injury (n=281)**		**0.0094**		**0.0036**
ORP	REF.		REF.	
LRP	1.17 (0.84-1.62)		1.14 (0.82-1.59)	
RARP	0.77 (0.57-1.05)		0.70 (0.50-0.98)	
**Vascular injury (n=141)**		0.8454		-
ORP	REF.		-	
LRP	1.08 (0.67-1.76)		-	
RARP	0.96 (0.63-1.48)		-	
**Other injury (n=127)**		0.6008		-
ORP	REF.		-	
LRP	0.86 (0.50-1.47)		-	
RARP	1.08 (0.69-1.68)		-	
**Stenosis (n=154)**		**<.0001**		0.1036
ORP	REF.		REF.	
LRP	0.71 (0.48-1.06)		0.76 (0.51-1.15)	
RARP	0.41 (0.28-0.60)		0.63 (0.41-0.97)	
**Sepsis (n=179)**		0.2016		0.1147
ORP	REF.		REF.	
LRP	1.09 (0.73-1.65)		1.08 (0.71-1.65)	
RARP	0.81 (0.56-1.18)		0.74 (0.49-1.13)	
**Embolism of phlebitis (n=350)**		**<.0001**		**0.0005**
ORP	REF.		REF.	
LRP	0.62 (0.47-0.81)		0.67 (0.51-0.89)	
RARP	0.48 (0.37-0.61)		0.58 (0.44-0.77)	
**Shock (n=228)**		**0.0020**		0.1883
ORP	REF.		REF.	
LRP	0.73 (0.51-1.03)		0.82 (0.57-1.17)	
RARP	0.57 (0.42-0.78)		0.72 (0.50-1.03)	
**Surgical wound dehiscence (n=447)**		**<.0001**		**<.0001**
ORP	REF.		REF.	
LRP	0.60 (0.48-0.76)		0.62 (0.49-0.79)	
RARP	0.34 (0.28-0.43)		0.35 (0.27-0.45)	
**Lymphocele (n=612)**		**<.0001**		**<.0001**

ORP	REF.		REF.	
LRP	0.77 (0.62-0.95)		0.93 (0.75-1.16)	
RARP	0.58 (0.48-0.71)		0.57 (0.46-0.70)	
**Urinary retention (n=1,175)**		**<.0001**		**<.0001**
ORP	REF.		REF.	
LRP	0.95 (0.82-1.12)		1.00 (0.85-1.17)	
RARP	0.64 (0.55-0.73)		0.71 (0.60-0.83)	

* Multivariable logistic regressions adjusted on age group, Charlson comorbidity index category, hospital type, hospital volume, Sars-Cov2 infection during hospital stay and lymph node dissection

[1] interaction between lymph node dissection and hospital type; [2] interaction between lymph node dissection and Charlson comorbidity index category; [3] interaction between hospital type and hospital volume; [4] interaction between hospital volume and lymph node dissection

ORP = Open radical prostatectomy / LRP = Laparoscopic radical prostatectomy / RARP = Robot-assisted radical prostatectomy
