# Supplementary material for: Comparison of short‐term complications after open, laparoscopic and robot‐assisted radical prostatectomy
Source: BJU Int. 2025 Nov 27;137(2):348–59. doi: 10.1111/bju.70076 (PMC12789849; doi:10.1111/bju.70076)
Supplement: Supplementary file 4 — Table S4. Association between the surgical approach and the occurrence of each subtype of adverse event during the initial hospital stay for RP for non‐metastatic prostate cancer (SNDS French national data from 1 January 2020 to 31 December 2021, N = 38 481), mixed‐effects logistic regression models. [file BJU-137-348-s007.pdf]

Supplementary table 4. Association between the surgical approach and the occurrence of each subtype of adverse event during the initial hospital stay for radical prostatectomy for non-metastatic prostate cancer (SNDS French national data from January 1, 2020, to December 31, 2021, n=38,481), mixed-effects logistic regression models

	ORP	LRP	RARP	p
**At least one adverse event (n=6,755)**	REF.	0.64 (0.57-0.71)	0.56 (0.49-0.63)	**<.0001**
**Intensive care unit admission (n=1,059)**	REF.	1.08 (0.76-1.55)	1.05 (0.76-1.46)	0.8996
**Overall complications (n=6,051)**	REF.	0.63 (0.57-0.70)	0.51 (0.45-0.57)	**<.0001**
**Haemorrhage (n=2,657)**	REF.	0.55 (0.47-0.64)	0.52 (0.44-0.62)	**<.0001**
**Infection (n=1,718)** *	REF.	0.45 (0.38-0.54)	0.36 (0.30-0.43)	**<.0001**
**Hernia (n=480)** [1]	REF.	1.19 (0.83-1.71)	0.97 (0.67-1.42)	0.3908
**Anastomotic leak or fistula (n=389)**	REF.	1.01 (0.75-1.37)	0.68 (0.49-0.94)	**0.0199**
**Bowel injury (n=291)**	REF.	1.66 (1.19-2.30)	0.66 (0.46-0.96)	**0.0036**
**Vesical or urethral injury (n=302)** [4]	REF.	2.66 (1.73-4.11)	1.87 (1.13-3.09)	**<.0001**
**Ureteric injury (n=211)**	REF.	1.16 (0.77-1.73)	0.63 (0.41-0.97)	**0.0072**
**Embolism of phlebitis (n=229)**	REF.	0.60 (0.41-0.90)	0.47 (0.31-0.73)	**0.0016**
**Shock (n=164)**	REF.	0.73 (0.47-1.13)	0.57 (0.36-0.90)	0.0540
**Surgical wound dehiscence (n=280)**	REF.	0.60 (0.43-0.84)	0.23 (0.16-0.34)	**<.0001**
**Lymphocele (n=306)**	REF.	0.71 (0.49-1.04)	0.69 (0.45-1.05)	0.1146
**Urinary retention (n=436)**	REF.	0.79 (0.60-1.03)	0.43 (0.30-0.60)	**<.0001**

ORP = Open radical prostatectomy / LRP = Laparoscopic radical prostatectomy / RARP = Robot-assisted radical prostatectomy. Estimations were adjusted on age, Charlson comorbidity index category, hospital type, hospital volume, Sars-Cov2 infection during hospital stay, lymph node dissection; center was considered as a random effect.

[1] interaction between lymph node dissection and hospital type; [2] interaction between lymph node dissection and Charlson comorbidity index category; [3] interaction between hospital type and hospital volume; [4] interaction between hospital volume and lymph node dissection

* log-linearity was not respected, age was categorized into four classes according to its distribution.
