# Supplementary material for: Comparison of short‐term complications after open, laparoscopic and robot‐assisted radical prostatectomy
Source: BJU Int. 2025 Nov 27;137(2):348–59. doi: 10.1111/bju.70076 (PMC12789849; doi:10.1111/bju.70076)
Supplement: Supplementary file 6 — Table S6. Subgroup description of the occurrence of adverse event during hospital stay for RP for non‐metastatic prostate cancer, in the overall population and according to surgical approach (SNDS French national data from 1 January 2020 to 31 December 2021, N = 38 481). [file BJU-137-348-s006.pdf]

Supplementary table 6. Subgroup description of the occurrence of adverse event during hospital stay for radical prostatectomy for non-metastatic prostate cancer, in the overall population and according to surgical approach (SNDS French national data from January 1, 2020, to December 31, 2021, n=38,841)

	LND (n = 23,076)				No LND (n=15,405)			
	**Overall**	**ORP**	**LRP**	**RARP**	**Overall**	**ORP**	**LRP**	**RARP**
**At least one adverse event**	4,042 (17.5%)	1,546 (26.2%)	933 (16.9%)	1,563 (13.4%)	2,713 (17.6%)	565 (27.8%)	795 (19.5%)	1353 (14.6%)
**ICU admission**	643 (2.8%)	245 (4.2%)	127 (2.3%)	271 (2.3%)	416 (2.7%)	90 (4.4%)	114 (2.8%)	212 (2.3%)
**In-hospital death**	24 (0.1%)	5 (0.1%)	5 (0.1%)	14 (0.1%)	6 (0.0%)	1 (0.0%)	2 (0.0%)	3 (0.0%)
**Overall complications**	3,615 (15.7%)	1,395 (23.7%)	850 (15.4%)	1,370 (11.7%)	2,436 (15.8%)	506 (24.9%)	730 (17.9%)	1,200 (12.9%)
Haemorrhage	1,638 (7.1%)	619 (10.5%)	354 (6.4%)	665 (5.7%)	1,019 (6.6%)	216 (10.6%)	252 (6.2%)	551 (5.9%)
Infection	1,080 (4.7%)	489 (8.3%)	230 (4.2%)	361 (3.1%)	638 (4.1%)	187 (9.2%)	182 (4.5%)	269 (2.9%)
Hernia	183 (0.8%)	41 (0.7%)	36 (0.7%)	106 (0.9%)	297 (1.9%)	25 (1.2%)	112 (2.7%)	160 (1.7%)
Evisceration or eventration	25 (0.1%)	6 (0.1%)	4 (0.1%)	15 (0.1%)	27 (0.2%)	7 (0.3%)	4 (0.1%)	16 (0.2%)
Anastomotic leak or fistula	244 (1.1%)	81 (1.4%)	69 (1.3%)	94 (0.8%)	145 (0.9%)	30 (1.5%)	53 (1.3%)	62 (0.7%)
Bowel injury	163 (0.7%)	49 (0.8%)	56 (1.0%)	58 (0.5%)	128 (0.8%)	20 (1.0%)	66 (1.6%)	42 (0.5%)
Vesical or urethral injury	127 (0.6%)	31 (0.5%)	46 (0.8%)	50 (0.4%)	175 (1.1%)	33 (1.6%)	65 (1.6%)	77 (0.8%)
Ureteric injury	122 (0.5%)	34 (0.6%)	37 (0.7%)	51 (0.4%)	89 (0.6%)	17 (0.8%)	37 (0.9%)	35 (0.4%)
Vascular injury	68 (0.3%)	12 (0.2%)	21 (0.4%)	35 (0.3%)	25 (0.2%)	3 (0.1%)	9 (0.2%)	13 (0.1%)
Other injury	53 (0.2%)	15 (0.3%)	8 (0.1%)	30 (0.3%)	27 (0.2%)	4 (0.2%)	10 (0.2%)	13 (0.1%)
Stenosis	49 (0.2%)	26 (0.4%)	9 (0.2%)	14 (0.1%)	54 (0.4%)	8 (0.4%)	21 (0.5%)	25 (0.3%)
Nerve lesion	11 (0.0%)	2 (0.0%)	3 (0.1%)	6 (0.1%)	5 (0.0%)	2 (0.1%)	1 (0.0%)	2 (0.0%)
Sepsis	55 (0.2%)	16 (0.3%)	20 (0.4%)	19 (0.2%)	37 (0.2%)	10 (0.5%)	11 (0.3%)	16 (0.2%)
Embolism or phlebitis	146 (0.6%)	65 (1.1%)	34 (0.6%)	47 (0.4%)	83 (0.5%)	27 (1.3%)	23 (0.6%)	33 (0.4%)
Shock	104 (0.5%)	43 (0.7%)	24 (0.4%)	37 (0.3%)	60 (0.4%)	13 (0.6%)	21 (0.5%)	26 (0.3%)
Surgical wound dehiscence	175 (0.8%)	77 (1.3%)	55 (1.0%)	43 (0.4%)	105 (0.7%)	36 (1.8%)	41 (1.0%)	28 (0.3%)

Lymphocele	262 (1.1%)	86 (1.5%)	69 (1.3%)	107 (0.9%)	44 (0.3%)	8 (0.4%)	8 (0.2%)	28 (0.3%)
Foreign bodies	3 (0.0%)	3 (0.1%)	0 (0.0%)	0 (0.0%)	5 (0.0%)	1 (0.0%)	3 (0.1%)	1 (0.0%)
Urinary retention	270 (1.2%)	120 (2.0%)	86 (1.6%)	64 (0.5%)	166 (1.1%)	43 (2.1%)	61 (1.5%)	62 (0.7%)

LND = Lymph node dissection / ORP = Open radical prostatectomy / LRP = Laparoscopic radical prostatectomy / RARP = Robot-assisted radical prostatectomy / ICU = Intensive Care Unit
