# Supplementary material for: Comparison of short‐term complications after open, laparoscopic and robot‐assisted radical prostatectomy
Source: BJU Int. 2025 Nov 27;137(2):348–59. doi: 10.1111/bju.70076 (PMC12789849; doi:10.1111/bju.70076)
Supplement: Supplementary file 7 — Table S7. Subgroup description of the occurrence of adverse event during initial hospital stay for RP for non‐metastatic prostate cancer and re‐hospitalisations starting within the 30 days after RP, in the overall population and according to surgical approach (SNDS French national data from 1 January 2020 to 31 December 2021, N = 38 481). [file BJU-137-348-s001.pdf]

Supplementary table 7. Subgroup description of the occurrence of adverse event during initial hospital stay for radical prostatectomy for non-metastatic prostate cancer and re-hospitalizations starting within the 30 days after radical prostatectomy, in the overall population and according to surgical approach (SNDS French national data from January 1, 2020, to December 31, 2021, n=38,841)

	LND (n = 23,076)				No LND (n=15,405)			
	**Overall**	**ORP**	**LRP**	**RARP**	**Overall**	**ORP**	**LRP**	**RARP**
**At least one adverse event**	5,460 (23.7%)	1,835 (31.1%)	1,286 (23.3%)	2339 (20.0%)	3,501 (22.7%)	663 (32.7%)	980 (24.0%)	1,858 (20.0%)
**ICU admission**	715 (3.1%)	259 (4.4%)	143 (2.6%)	313 (2.7%)	447 (2.9%)	92 (4.5%)	118 (2.9%)	237 (2.5%)
**In-hospital death**	34 (0.1%)	6 (0.1%)	9 (0.2%)	19 (0.2%)	9 (0.1%)	1 (0.0%)	2 (0.0%)	6 (0.1%)
**Overall complications**	5,057 (21.9%)	1,691 (28.7%)	1,210 (21.9%)	2,156 (18.5%)	3,239 (21.0%)	604 (29.8%)	919 (22.5%)	1,716 (18.5%)
Haemorrhage	2,062 (8.9%)	703 (11.9%)	444 (8.1%)	915 (7.8%)	1,279 (8.3%)	251 (12.4%)	308 (7.6%)	720 (7.7%)
Infection	1,898 (8.2%)	676 (11.5%)	444 (8.1%)	778 (6.7%)	1,125 (7.3%)	244 (12.0%)	314 (7.7%)	567 (6.1%)
Hernia	201 (0.9%)	43 (0.7%)	41 (0.7%)	117 (1.0%)	307 (2.0%)	26 (1.3%)	112 (2.7%)	169 (1.8%)
Evisceration or eventration	65 (0.3%)	10 (0.2%)	7 (0.1%)	48 (0.4%)	51 (0.3%)	8 (0.4%)	6 (0.1%)	37 (0.4%)
Anastomotic leak or fistula	414 (1.8%)	109 (1.8%)	116 (2.1%)	189 (1.6%)	269 (1.7%)	43 (2.1%)	88 (2.2%)	138 (1.5%)
Bowel injury	195 (0.8%)	52 (0.9%)	62 (1.1%)	81 (0.7%)	136 (0.9%)	21 (1.0%)	69 (1.7%)	46 (0.5%)
Vesical or urethral injury	169 (0.7%)	37 (0.6%)	63 (1.1%)	69 (0.6%)	213 (1.4%)	37 (1.8%)	76 (1.9%)	100 (1.1%)
Ureteric injury	172 (0.7%)	44 (0.7%)	47 (0.9%)	81 (0.7%)	109 (0.7%)	19 (0.9%)	42 (1.0%)	48 (0.5%)
Vascular injury	102 (0.4%)	25 (0.4%)	26 (0.5%)	51 (0.4%)	39 (0.3%)	4 (0.2%)	12 (0.3%)	23 (0.2%)
Other injury	81 (0.4%)	19 (0.3%)	12 (0.2%)	50 (0.4%)	46 (0.3%)	7 (0.3%)	15 (0.4%)	24 (0.3%)
Stenosis	87 (0.4%)	41 (0.7%)	20 (0.4%)	26 (0.2%)	67 (0.4%)	11 (0.5%)	25 (0.6%)	31 (0.3%)
Nerve lesion	12 (0.1%)	2 (0.0%)	3 (0.1%)	7 (0.1%)	6 (0.0%)	2 (0.1%)	1 (0.0%)	3 (0.0%)
Sepsis	114 (0.5%)	27 (0.5%)	34 (0.6%)	53 (0.5%)	65 (0.4%)	13 (0.6%)	19 (0.5%)	33 (0.4%)
Embolism or phlebitis	233 (1.0%)	86 (1.5%)	52 (0.9%)	95 (0.8%)	117 (0.8%)	30 (1.5%)	35 (0.9%)	52 (0.6%)
Shock	149 (0.6%)	52 (0.9%)	34 (0.6%)	63 (0.5%)	79 (0.5%)	15 (0.7%)	25 (0.6%)	39 (0.4%)
Surgical wound dehiscence	282 (1.2%)	115 (2.0%)	73 (1.3%)	94 (0.8%)	165 (1.1%)	53 (2.6%)	51 (1.3%)	61 (0.7%)

Lymphocele	537 (2.3%)	154 (2.6%)	151 (2.7%)	232 (2.0%)	75 (0.5%)	21 (1.0%)	13 (0.3%)	41 (0.4%)
Foreign bodies	4 (0.0%)	3 (0.1%)	0 (0.0%)	1 (0.0%)	5 (0.0%)	1 (0.0%)	3 (0.1%)	1 (0.0%)
Urinary retention	702 (3.0%)	217 (3.7%)	212 (3.8%)	273 (2.3%)	473 (3.1%)	87 (4.3%)	140 (3.4%)	246 (2.6%)

LND = Lymph node dissection / ORP = Open radical prostatectomy / LRP = Laparoscopic radical prostatectomy / RARP = Robot-assisted radical prostatectomy / ICU = Intensive Care Unit
