# Supplementary material for: Comparison of short‐term complications after open, laparoscopic and robot‐assisted radical prostatectomy
Source: BJU Int. 2025 Nov 27;137(2):348–59. doi: 10.1111/bju.70076 (PMC12789849; doi:10.1111/bju.70076)
Supplement: Supplementary file 9 — Table S9. Subgroup description of the occurrence of adverse event during initial hospital stay for RP for non‐metastatic prostate cancer, according to the annual activity of RP in the establishment (SNDS French national data from 1 January 2020 to 31 December 2021, N = 38 481). [file BJU-137-348-s005.pdf]

**Supplementary table 9.** Subgroup description of the occurrence of adverse event during initial hospital stay for radical prostatectomy for non-metastatic prostate cancer, according to the annual activity of RP in the establishment (SNDS French national data from January 1, 2020, to December 31, 2021, n=38,841)

| | **< 30 RP (n=1,857)** | | | | **>= 30 RP (n=36,624)** | | | |
|---|---|---|---|---|---|---|---|---|
| | **Overall** | **ORP** | **LRP** | **RARP** | **Overall** | **ORP** | **LRP** | **RARP** |
| **At least one adverse event** | 1,354 (23.5%) | 801 (28.8%) | 406 (19.4%) | 147 (16.6%) | 5,401 (16.5%) | 1,310 (25.5%) | 1,322 (17.6%) | 2,769 (13.8%) |
| **ICU admission** | 228 (4.0%) | 144 (5.2%) | 35 (1.7%) | 49 (5.5%) | 831 (2.5%) | 191 (3.7%) | 206 (2.7%) | 434 (2.2%) |
| **In-hospital death** | 7 (0.1%) | 5 (0.2%) | 1 (0.0%) | 1 (0.1%) | 23 (0.1%) | 1 (0.0%) | 6 (0.1%) | 16 (0.1%) |
| **Overall complications** | 1,212 (21.0%) | 709 (25.5%) | 390 (18.6%) | 113 (12.8%) | 4,839 (14.8%) | 1,192 (23.2%) | 1,190 (15.9%) | 2,457 (12.2%) |
| Haemorrhage | 519 (9.0%) | 314 (11.3%) | 144 (6.9%) | 61 (6.9%) | 2,138 (6.5%) | 521 (10.1%) | 462 (6.2%) | 1,155 (5.8%) |
| Infection | 378 (6.6%) | 265 (9.5%) | 92 (4.4%) | 21 (2.4%) | 1,340 (4.1%) | 411 (8.0%) | 320 (4.3%) | 609 (3.0%) |
| Hernia | 62 (1.1%) | 20 (0.7%) | 32 (1.5%) | 10 (1.1%) | 418 (1.3%) | 46 (0.9%) | 116 (1.5%) | 256 (1.3%) |
| Evisceration or eventration | 7 (0.1%) | 3 (0.1%) | 2 (0.1%) | 2 (0.2%) | 45 (0.1%) | 10 (0.2%) | 6 (0.1%) | 29 (0.1%) |
| Anastomotic leak or fistula | 91 (1.6%) | 52 (1.9%) | 27 (1.3%) | 12 (1.4%) | 298 (0.9%) | 59 (1.1%) | 95 (1.3%) | 144 (0.7%) |
| Bowel injury | 69 (1.2%) | 27 (1.0%) | 37 (1.8%) | 5 (0.6%) | 222 (0.7%) | 42 (0.8%) | 85 (1.1%) | 95 (0.5%) |
| Vesical or urethral injury | 27 (0.5%) | 6 (0.2%) | 16 (0.8%) | 5 (0.6%) | 275 (0.8%) | 58 (1.1%) | 95 (1.3%) | 122 (0.6%) |
| Ureteric injury | 38 (0.7%) | 13 (0.5%) | 20 (1.0%) | 5 (0.6%) | 173 (0.5%) | 38 (0.7%) | 54 (0.7%) | 81 (0.4%) |
| Vascular injury | 11 (0.2%) | 1 (0.0%) | 9 (0.4%) | 1 (0.1%) | 82 (0.3%) | 14 (0.3%) | 21 (0.3%) | 47 (0.2%) |
| Other injury | 9 (0.2%) | 5 (0.2%) | 2 (0.1%) | 2 (0.2%) | 71 (0.2%) | 14 (0.3%) | 16 (0.2%) | 41 (0.2%) |
| Stenosis | 30 (0.5%) | 19 (0.7%) | 9 (0.4%) | 2 (0.2%) | 73 (0.2%) | 15 (0.3%) | 21 (0.3%) | 37 (0.2%) |
| Nerve lesion | 5 (0.1%) | 2 (0.1%) | 2 (0.1%) | 1 (0.1%) | 11 (0.0%) | 2 (0.0%) | 2 (0.0%) | 7 (0.0%) |
| Sepsis | 15 (0.3%) | 9 (0.3%) | 6 (0.3%) | 0 (0.0%) | 77 (0.2%) | 17 (0.3%) | 25 (0.3%) | 35 (0.2%) |
| Embolism or phlebitis | 62 (1.1%) | 38 (1.4%) | 17 (0.8%) | 7 (0.8%) | 167 (0.5%) | 54 (1.1%) | 40 (0.5%) | 73 (0.4%) |
| Shock | 46 (0.8%) | 30 (1.1%) | 15 (0.7%) | 1 (0.1%) | 118 (0.4%) | 26 (0.5%) | 30 (0.4%) | 62 (0.3%) |
| Surgical wound dehiscence | 59 (1.0%) | 37 (1.3%) | 17 (0.8%) | 5 (0.6%) | 221 (0.7%) | 76 (1.5%) | 79 (1.1%) | 66 (0.3%) |

| | | | | | | | | |
|---|---|---|---|---|---|---|---|---|
| Lymphocele | 40 (0.7%) | 19 (0.7%) | 14 (0.7%) | 7 (0.8%) | 266 (0.8%) | 75 (1.5%) | 63 (0.8%) | 128 (0.6%) |
| Foreign bodies | 1 (0.0%) | 1 (0.0%) | 0 (0.0%) | 0 (0.0%) | 7 (0.0%) | 3 (0.1%) | 3 (0.0%) | 1 (0.0%) |
| Urinary retention | 120 (2.1%) | 64 (2.3%) | 48 (2.3%) | 8 (0.9%) | 316 (1.0%) | 99 (1.9%) | 99 (1.3%) | 118 (0.6%) |

*ORP = Open radical prostatectomy / LRP = Laparoscopic radical prostatectomy / RARP = Robot-assisted radical prostatectomy / ICU = Intensive Care Unit*
