# Supplementary material for: Comparison of short‐term complications after open, laparoscopic and robot‐assisted radical prostatectomy
Source: BJU Int. 2025 Nov 27;137(2):348–59. doi: 10.1111/bju.70076 (PMC12789849; doi:10.1111/bju.70076)
Supplement: Supplementary file 10 — Table S10. Subgroup description of the occurrence of adverse event during initial hospital stay for RP for non‐metastatic prostate cancer, according to the annual activity of RP in the establishment (SNDS French national data from 1 January 2020 to 31 December 2021, N = 38 481). [file BJU-137-348-s010.pdf]

Supplementary table 10. Subgroup description of the occurrence of adverse event during initial hospital stay for radical prostatectomy for non-metastatic prostate cancer, according to the annual activity of RP in the establishment (SNDS French national data from January 1, 2020, to December 31, 2021, n=38,841)

	< 60 RP (n=1,857)				>= 60 RP (n=36,624)			
	**Overall**	**ORP**	**LRP**	**RARP**	**Overall**	**ORP**	**LRP**	**RARP**
**At least one adverse event**	3,459 (21.7%)	1,465 (28.0%)	1,017 (19.7%)	977 (17.5%)	3,296 (14.6%)	646 (24.1%)	711 (16.0%)	1,939 (12.6%)
**ICU admission**	543 (3.4%)	247 (4.7%)	108 (2.1%)	188 (3.4%)	516 (2.3%)	88 (3.3%)	133 (3.0%)	295 (1.9%)
**In-hospital death**	19 (0.1%)	6 (0.1%)	5 (0.1%)	8 (0.1%)	11 (0.0%)	0 (0.0%)	2 (0.0%)	9 (0.1%)
**Overall complications**	3,124 (19.6%)	1,313 (25.1%)	965 (18.7%)	846 (15.2%)	2,927 (13.0%)	588 (21.9%)	615 (13.9%)	1,724 (11.2%)
Haemorrhage	1,433 (9.0%)	580 (11.1%)	368 (7.1%)	485 (8.7%)	1,224 (5.4%)	255 (9.5%)	238 (5.4%)	731 (4.8%)
Infection	833 (5.2%)	458 (8.7%)	225 (4.4%)	150 (2.7%)	885 (3.9%)	218 (8.1%)	187 (4.2%)	480 (3.1%)
Hernia	204 (1.3%)	41 (0.8%)	90 (1.7%)	73 (1.3%)	276 (1.2%)	25 (0.9%)	58 (1.3%)	193 (1.3%)
Evisceration or eventration	21 (0.1%)	8 (0.2%)	6 (0.1%)	7 (0.1%)	31 (0.1%)	5 (0.2%)	2 (0.0%)	24 (0.2%)
Anastomotic leak or fistula	215 (1.3%)	79 (1.5%)	76 (1.5%)	60 (1.1%)	174 (0.8%)	32 (1.2%)	46 (1.0%)	96 (0.6%)
Bowel injury	166 (1.0%)	54 (1.0%)	86 (1.7%)	26 (0.5%)	125 (0.6%)	15 (0.6%)	36 (0.8%)	74 (0.5%)
Vesical or urethral injury	156 (1.0%)	50 (1.0%)	74 (1.4%)	32 (0.6%)	146 (0.6%)	14 (0.5%)	37 (0.8%)	95 (0.6%)
Ureteric injury	93 (0.6%)	30 (0.6%)	39 (0.8%)	24 (0.4%)	118 (0.5%)	21 (0.8%)	35 (0.8%)	62 (0.4%)
Vascular injury	34 (0.2%)	6 (0.1%)	18 (0.3%)	10 (0.2%)	59 (0.3%)	9 (0.3%)	12 (0.3%)	38 (0.2%)
Other injury	29 (0.2%)	13 (0.2%)	7 (0.1%)	9 (0.2%)	51 (0.2%)	6 (0.2%)	11 (0.2%)	34 (0.2%)
Stenosis	57 (0.4%)	28 (0.5%)	18 (0.3%)	11 (0.2%)	46 (0.2%)	6 (0.2%)	12 (0.3%)	28 (0.2%)
Nerve lesion	10 (0.1%)	3 (0.1%)	3 (0.1%)	4 (0.1%)	6 (0.0%)	1 (0.0%)	1 (0.0%)	4 (0.0%)
Sepsis	44 (0.3%)	17 (0.3%)	16 (0.3%)	11 (0.2%)	48 (0.2%)	9 (0.3%)	15 (0.3%)	24 (0.2%)
Embolism or phlebitis	139 (0.9%)	69 (1.3%)	39 (0.8%)	31 (0.6%)	90 (0.4%)	23 (0.9%)	18 (0.4%)	49 (0.3%)
Shock	103 (0.6%)	43 (0.8%)	35 (0.7%)	25 (0.4%)	61 (0.3%)	13 (0.5%)	10 (0.2%)	38 (0.2%)
Surgical wound dehiscence	160 (1.0%)	76 (1.5%)	61 (1.2%)	23 (0.4%)	120 (0.5%)	37 (1.4%)	35 (0.8%)	48 (0.3%)

Lymphocele	120 (0.8%)	43 (0.8%)	35 (0.7%)	42 (0.8%)	186 (0.8%)	51 (1.9%)	42 (0.9%)	93 (0.6%)
Foreign bodies	5 (0.0%)	4 (0.1%)	1 (0.0%)	0 (0.0%)	3 (0.0%)	0 (0.0%)	2 (0.0%)	1 (0.0%)
Urinary retention	238 (1.5%)	103 (2.0%)	97 (1.9%)	38 (0.7%)	198 (0.9%)	60 (2.2%)	50 (1.1%)	88 (0.6%)

ORP = Open radical prostatectomy / LRP = Laparoscopic radical prostatectomy / RARP = Robot-assisted radical prostatectomy / ICU = Intensive Care Unit
